# Enzyme promiscuity in natural environments: alkaline phosphatase in the ocean

**DOI:** 10.1038/s41396-021-01013-w

**Published:** 2021-05-28

**Authors:** Abhishek Srivastava, Daniel E. M. Saavedra, Blair Thomson, Juan A. L. García, Zihao Zhao, Wayne M. Patrick, Gerhard J. Herndl, Federico Baltar

**Affiliations:** 1 Department of Functional and Evolutionary Ecology, University of Vienna, Vienna, Austria; 2 Department of Marine Science, University of Otago, Dunedin, New Zealand; 3 School of Biological Sciences, Victoria University of Wellington, Kelburn, New Zealand; 4 NIOZ, Department of Marine Microbiology and Biogeochemistry, Royal Netherlands Institute for Sea Research, Utrecht University, Texel, The Netherlands

**Keywords:** Microbial biooceanography, Biogeochemistry, Microbial ecology

## Abstract

Alkaline phosphatase (APase) is one of the marine enzymes used by oceanic microbes to obtain inorganic phosphorus (P_i_) from dissolved organic phosphorus to overcome P-limitation. Marine APase is generally recognized to perform P-monoesterase activity. Here we integrated a biochemical characterization of a specific APase enzyme, examination of global ocean databases, and field measurements, to study the type and relevance of marine APase promiscuity. We performed an in silico mining of *phoA* homologs, followed by de novo synthesis and heterologous expression in *E. coli* of the full-length gene from *Alteromonas mediterranea*, resulting in a recombinant PhoA. A global analysis using the TARA Oceans, Malaspina and other metagenomic databases confirmed the predicted widespread distribution of the gene encoding the targeted PhoA in all oceanic basins throughout the water column. Kinetic assays with the purified PhoA enzyme revealed that this enzyme exhibits not only the predicted P-monoester activity, but also P-diesterase, P-triesterase and sulfatase activity as a result of a promiscuous behavior. Among all activities, P-monoester bond hydrolysis exhibited the highest catalytic activity of APase despite its lower affinity for phosphate monoesters. APase is highly efficient as a P-monoesterase at high substrate concentrations, whereas promiscuous activities of APase, like diesterase, triesterase, and sulfatase activities are more efficient at low substrate concentrations. Strong similarities were observed between the monoesterase:diesterase ratio of the purified PhoA protein in the laboratory and in natural seawater. Thus, our results reveal enzyme promiscuity of APase playing potentially an important role in the marine phosphorus cycle.

## Introduction

Microbes are the engines driving the biochemical cycles on Earth [1]. Most biogeochemical models are based on the catalytic transformation of biomolecules caused by the activity of microbial enzymes, relying on the assumption that these enzymes perform only a single function [2–6]. However, some enzymes across protein families can have “promiscuous” activities [7, 8]. One of these promiscuous enzymes is alkaline phosphatase (APase), primarily a P-monoesterase enzyme [9], which in *Escherichia coli* can act as a promiscuous P-diesterase and sulfatase [10, 11]. Furthermore, evidence of *E. coli* APase acting as a hydrogenase also comes from its capability to oxidize phosphite into phosphate and molecular hydrogen [12]. Sensu stricto definition of enzyme promiscuity may pertain to secondary reactions conducted by an enzyme that are physiologically irrelevant and may not contribute to organismal fitness in an environment [13]. However, in a marine setting, where many different potential substrates are available to an enzyme like APase, promiscuous activities may play a role that could be physiologically relevant to the host microbes. Examples of extracellular peptidases in a marine environment support this concept where a commonly available substrate can be hydrolyzed by an array of specific enzymes [14].

Here we tested the promiscuity of a common APase of marine origin, i.e., from the Gammaproteobacterium *Alteromonas mediterranea* strain DE (deep ecotype). We have selected *phoA* from *Alteromonas* due to the global distribution of this genus and their ubiquitous presence in oligotrophic surface waters, the deep sea and in coastal areas [15], reaching high relative abundances (up to 30% of total prokaryotic abundance) especially associated with phytoplankton blooms [16]. Since APase can act as a promiscuous P-diesterase and sulfatase in *E. coli* [10, 11], we hypothesized that marine microbes could produce promiscuous APase (monoesterase), which also exhibits P-diesterase, P-triesterase, and sulfatase activities. If confirmed, the promiscuity of marine APase will potentially expand the role of APase within the phosphorus (P) cycle by targeting a higher diversity of potential substrate types.

## Methods

We performed first an in silico mining of *phoA* homologs, followed by de novo synthesis and heterologous expression in *E. coli* of the full-length (1467-bp) gene from *A. mediterranea* strain DE, resulting in a soluble recombinant PhoA (Supplementary Figs. 1 and 2). This purified PhoA was used in our enzymatic assays for further testing of enzymatic properties. Further details are given below.

### Evaluation of gene abundance in the global ocean

**Fig. 1:**
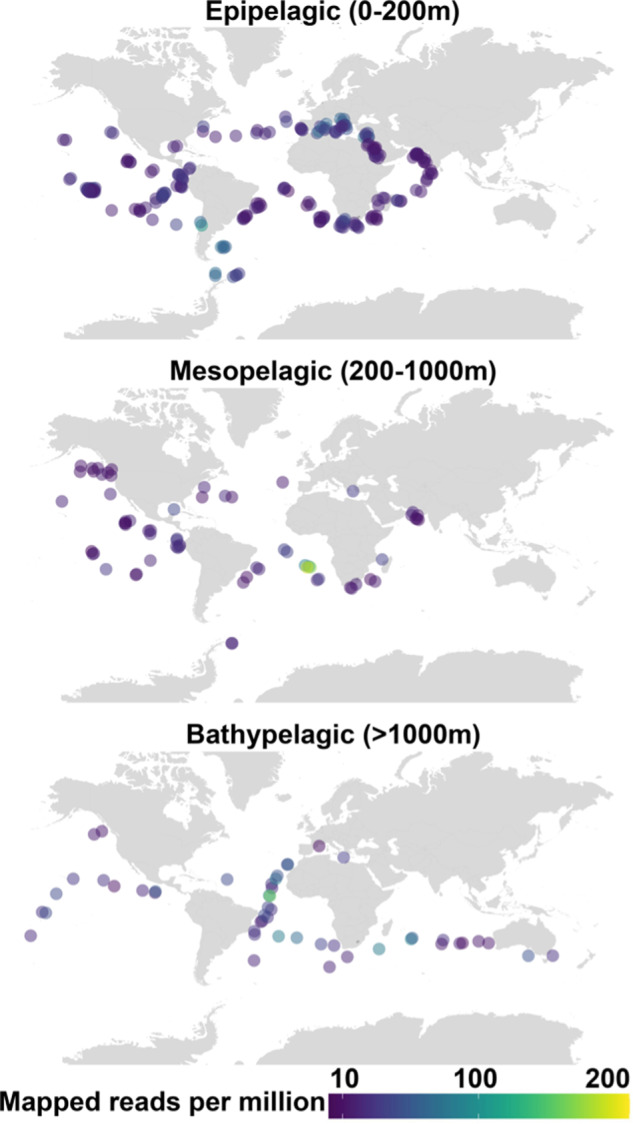
Global distribution of the homologs of *pho*A from *Alteromonas mediterranea* strain DE in the global ocean. The colour of the dots represents the mapped reads per million (see Methods for details). Three subplots are presented, depicting different depth layers: i.e., Epipelagic (0–200 m depth) in the top panel, Mesopelagic (200–1000 m depth) in the middle panel, and Bathypelagic (>1000 m depth) in the bottom panel.

The metagenomic dataset of marine microbes was downloaded from The National Center for Biotechnology Information portal using accession numbers tabulated in Supplementary Table 1. The quality of the metagenomic reads were check using fastQC and trimmed with AdaptorRemoval v2 with default setting [17]. Qualified reads were kept for downstream assembly. The assembly for each metagenomic library was carried out using Megahit v.1.1.2 [18] (k list: 21, 29, 39, 59, 79, 99, 119, 141). Putative genes were predicted on contigs only with length that exceeded 200-bp using Prodigal v.2.6.3 (under option “-p meta” metagenomic mode) [19]. The number of reads mapped to genes and coverage in each metagenome was evaluated by mapping reads back to the predicted gene sequences using Burrows–Wheeler Aligner package (bwa mem, 0.7.16a). The number of mapped reads to each gene was extracted from sam file using BBtools (pileup.sh) [20]. Gene abundance was calculated using the following equation that considers reads per million mapped reads: RPM = 1 M × (mapped reads/gene length)/(sum of mapped reads/gene length). PhoA-like sequences were identified by searching predicted genes against the curated HMM model PF00245 downloaded from Pfam (https://pfam.xfam.org/) using hmmscan (HMMER 3.2.1, http://hmmer.org/). PhoA-like sequences with domain coverage >40% and *E*-value <1 × 10^−12^ were retained for analysis. The corresponding gene abundance of positive *phoA*-like sequences was extracted from the results of the previous gene abundance evaluation. These data were fed into R program v.3.6.1 using the ggplot2 graphics package for visualization. Color scale was used to show the *phoA* homolog abundances in the global oceans (Fig. 1).

### Gene synthesis and cloning

*phoA* gene sequences from *Alteromonas mediterranea* strain DE (MADE_1013045) were used for de novo biosynthesis. Plasmid MADE_1013045_pET28a was ordered with pelB signal for periplasmic expression and with the HIS6-Prescissio3C cleavage site on N-terminus synthetically from Twist Bioscience, already cloned into their expression vector. The gene was then amplified with forward primer binding to the Prescission 3C recognition site (5′- ctggaagttctgttccaggg - 3′) and reverse primer binding to a stretch just after the STOP codon that was included on the synthetic construct for this purpose (5′- ccccagaacatcaggttaatggcg- 3′). These PCR products were then cloned into the pETM-14 vector bearing the N-terminal His6 and PreScission 3C cleavage site for cytoplasmic expression via RecA-mediated SLIC strategy as described by Scholz et al. [21].

### Protein expression

Recombinant proteins were expressed in *E. coli* strain Bl21(DE3) transformed with MADE1013045_pETM14 construct by autoinduction (AI) with Terrific Broth (TB) and 1.5% lactose at 25 °C for 24 h. Frozen cells from 500 ml of expression culture were thawed and re-suspended in 100 ml lysis buffer and sonicated for 12 min. Purification was conducted by deploying Histrap-based immobilized metal affinity chromatography. We concluded that the majority of MADE1013045 is soluble after His-tag cleavage and yields ~50-kDa protein (Supplementary Figs. 1 and 2 and Supplementary Tables 2 and 3).

Expression construct MADE1013045_pETM-14 with secretory signal was transformed into *E. coli* strain Bl21(DE3). This construct without secretory signal was transformed into shuffle cells. Positive clones were selected using kanamycin antibiotic. Recombinant proteins were expressed by AI with TB and 1.5% lactose at 18 or 25 °C for 24 h. Cells from 500 ml of expression culture were re-suspended in 100 ml lysis buffer and sonicated for 12 min. From this, a whole cell lysate sample was taken. The intracellular soluble fraction was isolated by centrifugation at 14,000 × *g*, at 4 °C for 30 min and 100 ml were loaded onto a 5 ml Nickel-Histrap column, washed with 10 or 12 column volumes (CV) of wash buffer (I and II), and eluted in 8 CV elution buffer. The lysis buffer contained: 20 mM Tris pH 7.5, 300 mM NaCl, 20 mM Imidazol, 0.5 mM tris(2-carboxyethyl)phosphine (TCEP), protease inhibitor, 2 μl benzonase/ml buffer. Samples of 20 μl of the elution fraction were taken, mixed with loading dye and loaded on a 12% SDS-PAGE together with whole cell lysate, intracellular soluble fraction, supernatant, and flowthrough samples (5 μl of 1:10 dilution loaded) and ran with 1 × SDS buffer for 50 min. The elution buffer contained: 20 mM Tris, pH 7.5, 300 mM NaCl, 400 mM Imidazol, 0.5 mM TCEP. High purity PhoA protein (450 μl, MADE1013045) with 10.6 mg/ml concentration (208 μM) were purified by size exclusion chromatography (Supplementary Fig. 2) and were stored at −80 °C.

### Determination of enzyme kinetic parameters of the PhoA protein under controlled laboratory conditions

APase (PhoA) from *Alteromonas mediterranea* strain DE was tested for monoesterase, diesterase, triesterase, and sulfatase activity using p-nitrophenol substrates (p-nitrophenyl phosphate disodium salt hexahydrate, Bis(p-nitrophenyl) phosphate sodium salt, Paraoxon-methyl, and 4-nitrophenyl sulfate potassium salt) from Sigma-Aldrich (Merck KGaA, Darmstadt, Germany). Activity of PhoA was measured based on an assay that relies upon hydrolyzing capability of the enzyme that releases chromogenic product p-nitrophenol from the above stated substrates and the product is then detected measuring the absorbance at 405 nm. A description of the mono-, di-, tri-, and sulfo-esterase activities measurement by chromogenic product detection methods can be found elsewhere [22–24]. For the standard curve, p-nitrophenol (ACROS Organics, Fisher Scientific GmbH, Austria) was used. All reactions were carried out in a 100 mM Tris buffered solution (pH 8.2) at 24 °C with the addition of magnesium chloride (100 µM) for higher activity. Protein and substrate concentrations were chosen depending on the substrate as summarized here: (1) for the monoesterase activity: 1 nM of enzyme and 0.001–100 mM of substrate (*p*-Nitrophenylphosphate); (2) for the diesterase activity: 10 nM enzyme and 0.00025–2.5 mM of Bis-*p*-nitrophenylphosphate; (3) for the triesterase activity: 10 nM enzyme and 0.00002–0.02 mM of Paraoxon-methyl; (4) for sulfatase activity: 6.9 nM enzyme and 0.000005–0.5 mM of 4-nitrophenylsulfate. The absorption values were measured in technical triplicates with a microplate reader (TECAN, Infinite 200 PRO) in volumes of 300 µl at 405 nm wavelength and data were recorded for 2 h at 2-min intervals. Blanks were run for each substrate and substrate concentration without the addition of any enzyme and subtracted to the corresponding substrate. The concentration of product (p-nitrophenol) was determined by comparing the relative absorbance to the absorbance of a standard curve of known standard concentrations (ranging between 0 and 250 µM). Biological triplicates were used for mono-, di-, and triesterase and biological quadruplets for sulfatases. Initial rate constants were calculated from each replicate independently using the initial linear region of the kinetics experiment and fitted into a linear regression. Michaelis–Menten kinetics and *k*_cat_ value evaluations with the corresponding enzyme concentration were obtained using R v.3.6.1 and script [25].

### Enzymatic activity assay of the alkaline phosphatase (monoesterase) and diesterase activity in South Pacific coastal waters

A biweekly coastal sampling was carried out at the University of Otago’s Portobello Marine Laboratory, situated on the Otago Harbour, Dunedin, New Zealand for over 20 months, between October 2017 and June 2019. The laboratory is based at the outer basin with waters similar in composition to coastal seawater with short residence times in exchanging with the open sea [26, 27]. Seawater samples were taken in triplicate from 1 m depth off the marine laboratory’s wharf, which extends into a deep tidal channel using a sampler that opens and closes at depth to avoid surface biofilms. Samples were always taken 1 h before high tide fortnightly to maximize the coastal influence in each sample and processed immediately.

Extracellular enzymatic activities were assessed based on hydrolysis of fluorogenic substrate analogs [28]. The fluorogenic substrates 4-methylumbelliferyl (MUF)-phosphate and Bis 4-MUF phosphate were used to assess the APase and phosphodiesterase activities, respectively. Saturating substrate concentrations of 100 μM were used based on preestablished kinetics. As a consequence of adding saturating concentrations, this technique yields potential maximum rates. Greiner Bio-one 96-well non-binding microplates were filled with six technical replicates of the fluorogenic substrates (10 μl of 3 mM MUF phosphate) and seawater (290 μl) reaching final concentrations of 100 μM to make up 300 μl reactions. Standards were prepared using 0.22-μm-filtered seawater. Plates were read in a Spectramax M2 spectrofluorometer (Molecular Devices, USA) with excitation and emission wavelengths of 365 and 445 nm, respectively, both before and after 3 h incubations. The detection limit was 3.0 fmol/well in the 300 μl FITC 96 wells. Six replicates without substrate addition served as blanks in each plate. All incubations were performed in the dark at in situ seawater temperature.

## Results and discussion

Our global analysis using the TARA Oceans, Malaspina, and other metagenomic databases confirmed the predicted widespread distribution of the gene encoding the targeted PhoA (DE) homologs from *Alteromonas* in the ocean and at all depths (i.e., epi-, meso-, and bathypelagic) (Fig. 1 and Supplementary Table 1). Yet, finding homologs of a gene in a database does not necessarily translate into having expressed proteins that carry out a specific function. An important next step will be to characterize the APase enzymes from other abundant marine bacteria, to test whether the promiscuity observed in PhoA of *A. mediterranea* (see below details on enzyme kinetic data) is a conserved feature of marine APases.

Apart from in silico mining of *phoA* homologs, the purified PhoA was generated and used in our enzymatic assays. Kinetic enzymatic assays revealed that PhoA showed not only phosphatase (monoesterase) activity but also P-diesterase, P-triesterase, and sulfatase activities (Table 1 and Supplementary Fig. 3). Hence, our results suggest that the pool of dissolved organic phosphorus (DOP) compounds with phospho mono-, di-, and triester bonds is accessible through the use of a single, multifunctional marine APase enzyme.

**Table 1:** Kinetic parameters of PhoA at 24 °C, in 100 mM Tris buffered solution (pH 8.2) with 100 µM magnesium chloride.

Substrate	*k*_cat_ (s^−1^)	*K*_M_ (µmol l^−1^)	*k*_cat_/*K*_M_ (µmol l^−1^ s^−1^)
P-monoesterase	0.5 ± 0.03	94 ± 35	(4.8 ± 0.8) × 10^−3^
P-diesterase	(1.7 ± 0.1) × 10^−2^	0.3 ± 0.1	(5.5 ± 0.8) × 10^−2^
P-triesterase	(2.5 ± 0.1) × 10^−2^	(1.5 ± 0.4) × 10^−2^	1.7 ± 0.3

Data are reported as the mean ± standard deviation of curve fitting.

*k*_*cat*_ turnover rate (*V*_max_ divided by enzyme concentration), *K*_*M*_ Michaelis–Menten constant, *k*_*cat*_*/K*_*M*_ catalytic efficiency.

The turnover number (*k*_cat_) and the Michaelis–Menten constant (*K*_M_) of APase for monoester substrate were always more than one order of magnitude higher in comparison to P-diester, P-triester, and sulfate-ester substrates (Table 1). P-monoester bond hydrolysis exhibited the highest catalytic activity of APase (highest phosphate releasing capability) despite its lower affinity for phosphate monoester substrate. The Michaelis–Menten constant of P-monoesterase (*K*_M_ = 90 µM) is orders of magnitude higher than the 0–45 nM range expected for hydrolysable P-monoesters in the open ocean [29]. This relatively higher *K*_M_, together with the higher *k*_cat_ of P-monoesterase, may indicate a preferential role of P-monoesterase during high organic P concentrations conditions (such as those found during phytoplankton blooms or in sinking of marine snow particles, environments particularly suited for *Alteromonas*). These results are consistent with previous report indicating a strong potential of marine P-monoesterase activity to rapidly respond to sporadic pulses of organic matter [30].

**Fig. 2:**
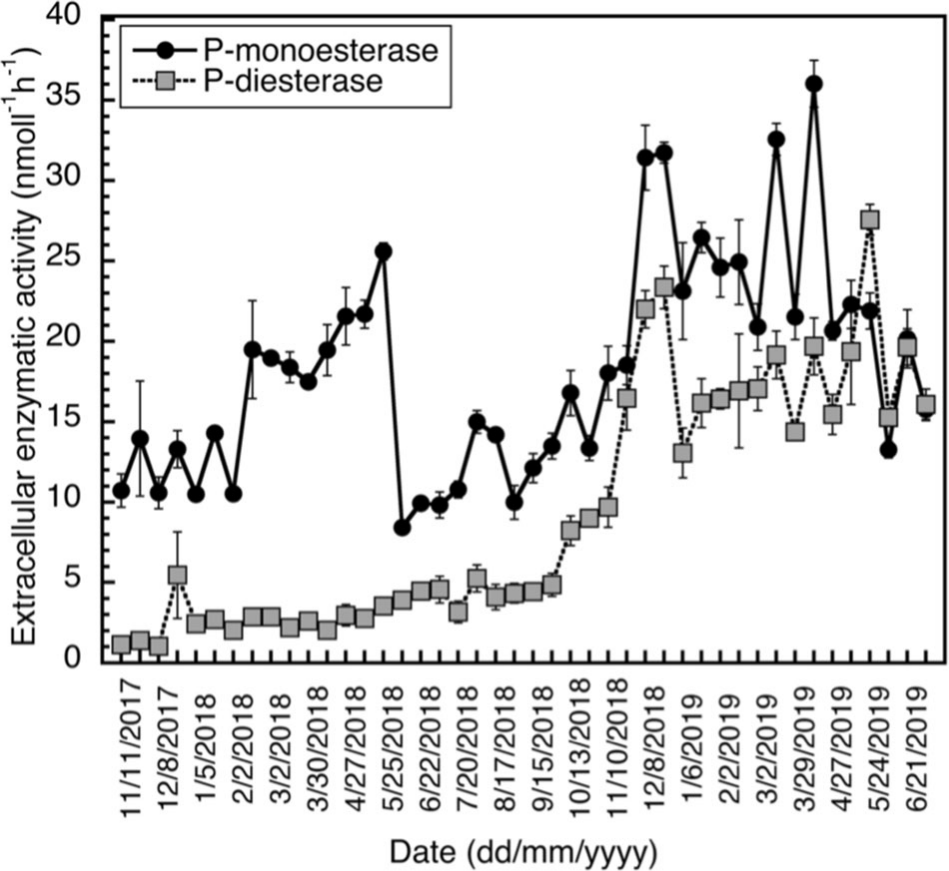
APase and diesterase activity data from South Pacific Ocean collected biweekly for 20 months. Phosphatase and diesterase activities were measured via fluorometric detection of enzymatically released 4-methylumbelliferyl.

The catalytic efficiency (*k*_cat_/*K*_M_) for di- and especially triesterase shows that the enzyme will hydrolyze these substrates more efficiently than P-monoesters at lower concentrations. Therefore, it is possible that the P-diesterase and P-triesterase activities of the enzyme may become particularly crucial during oligotrophic (low DOP) conditions. Remarkably, the obtained *K*_M_ values for diesterase and triesterase are comparable with concentrations of natural substrates in the oceanic water column (i.e., 0–45 nM for diesterase and <1 nM for triesterase) [29, 31], indicating that this enzyme may act as a multifunctional enzyme in the native marine environment. Although there is evidence that kinetics parameters measured in vitro are useful predictors of in vivo functionality [32], the conditions in the experiment do not completely reflect in situ conditions. Interestingly, we detected a continuous presence of P-monoesterase and P-diesterase activities, and significant correlation (*r* = 0.667, *p* value < 0.005) of these activities, in a high resolution (biweekly) monitoring over a period of almost 2 years in natural communities from the South Pacific Ocean (Fig. 2). Intriguingly, the monoesterase:diesterase ratio ranged between 2 and 10 (Fig. 2). When the diesterase activity was low (during the first 6–8 months) and quite stable, the monoesterase:diesterase was near 10, consistent with the ca. 10:1 monoesterase to diesterase promiscuous activity we detected in our PhoA protein (Table 1). After that period, the diesterase activity increased drastically, reducing the monoesterase:diesterase ratio. Most of the diesterase activity recorded in the early period (of low and stable diesterase activity) might be a background result of the promiscuous activity of APase, whereas the following increase in diesterase activities (in general and relative to monoesterase activity) might indicate the active generation of dedicated specific diesterase enzymes. However, more evidence would be required to support this hypothesis, since it is also possible that the patterns observed might be associated to changes in the diversity of microbes and enzymes during the sampling period rather than promiscuous activity. Nevertheless, the similarities observed between the monoesterase:diesterase ratio of the purified PhoA protein and of the natural seawater activities are remarkable during the period of low diesterase activity.

Our results also indicate that marine microbes might also use P-triesters as a source of P via APase activity. This represents a previously unrecognized source of P for APase (which also provides an ecological advantage), as well as a hitherto unknown potential sink of synthetic organophosphate esters. Orthophosphate diester compounds (e.g., deoxyribonucleic acids and lipids) are organic in origin and can account for up to 17% of the DOP [33], whereas phosphate triester compounds are also synthetic (e.g., plasticizers and pesticides) [32]. In addition, DNA damage in a cell may be due to appearance of alkyl phosphotriester lesions caused by endogenous or exogenous alkylating agents, suggesting biological origin of triesters [34, 35]. P-triesterase activity (promiscuous or not) may be important for cells as a detoxification mechanism since P-triesters also occur in DNA. Interestingly, APase displayed its lowest Michaelis–Menten constant (*K*_M_ = 15 nM) for these P-triester substrates, suggesting that their hydrolysis could be achieved in the native environment (since the concentration of these compounds in the ocean is <1 nM) [31]. Detoxification of synthetic organophosphates could also be an important role for APase that might require more attention, particularly bearing in mind the importance of anthropogenic pollution in the ocean.

The promiscuous sulfatase activity of APase was weak but detectable (*K*_m_ was 1.0 nM and the *k*_cat_/*K*_m_ was 16 µmol l^−1^ s^−1^). Marine sulfatases allow the microbes to utilize S-containing substrates such the sulfonated polysaccharides and lipopolysaccharides from algae and bacteria, respectively [36]. Considering the high diversity of marine sulfatases [37] and high abundance of sulfate in ocean [38], low level of APase’s sulfatase activity is prima facie negligible. Nevertheless, based upon our observation the sulfatase activity of APase is possible if the substrate is encountered.

**Fig. 3:**
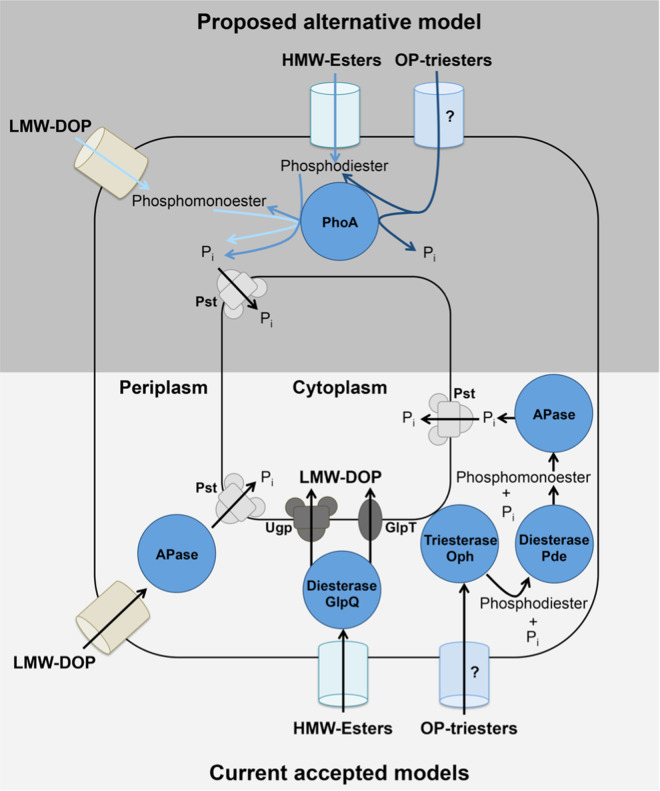
Model depicting an alternative P acquisition strategy and short-circuiting of the phosphorus cycle at the marine bacterial alkaline phosphatase nucleating point. Upper gray panel shows an alternative model based upon alkaline phosphatase promiscuity found in our study. Lower beige panel shows the classical P acquisition model based upon Dyhrman et al., Larson et al., van Veen, Parthasarathy et al. [45–48]. APase alkaline phosphatase, Oph phosphotriesterase or organophosphate hydrolase, GlpQ glycerophosphodiester phosphodiesterase, GlpT sn-glycerol-3-phosphate transporter, HMW-Esters high molecular weight esters, LMW-DOP low molecular weight dissolved organic phosphorus, OP-triesters organophosphate-triesters, Pde phosphodiesterase, PhoA alkaline phosphatase (PhoA family), P_i_ inorganic phosphorus, Pst high-affinity inorganic phosphate transporter, Ugp sn-glycerol 3-phosphate ABC transporter.

Collectively, our findings reveal a diverse role of microbial APase in P acquisition and DOP remineralization in the marine environment. It is important to note that three APase gene families are categorically known in prokaryotes i.e., PhoA, PhoD, and PhoX, where latter two are predicted to be more abundant in ocean than PhoA [39]. PhoD and PhoX have specificity toward diester substrate in addition to monoester substrate, whereas PhoA has specificity toward monoester substrate only [39–43]. In addition, there is no reported evidence of PhoD and PhoX phosphatases exhibiting triesterase and sulfatase activity. Thus, it would be now worth testing PhoD and PhoX for wider substrate activity. Several models are available explaining prokaryotic transport systems for phosphate import into the cell and utilization of phosphate via dedicated enzyme systems [44–47] (Fig. 3). Periplasmic enzymes like APase, P-diesterase (GlpQ, Pde), and P-triesterase (Oph) catalyze the hydrolysis of P-monoester, P-diester, and P-triester containing compounds, respectively. Current models on the utilization of external DOP compounds by bacteria are based on non-promiscuous periplasmic enzymes [44–47]. In these models, dephosphorylation of organophosphate molecules within the periplasmic space via PhoA has been shown to lead to an enrichment of P_i_ when only monoesterase activity was tested [48]. Hydrolyzed products like P_i_ and ester-bond containing molecules are subsequently transported via ATP-binding cassette transport systems, i.e., Pst and Ugp systems, respectively (Fig. 3). The major facilitator superfamily (mfs) transporter-like monomeric permease (GlpT) additionally imports glycerol 3-phosphate, which is the hydrolysis product of glycerophosphodiester phosphodiesterase [46]. In contrast, in the light of our findings an alternative (“enzyme promiscuity”) model can be developed where P-diester and P-triester compounds can also be processed that extends the relevance of APase promiscuity in the marine P cycle (Fig. 3). In the event of cell-lysis, cell-free enzyme devoid of cellular genetic regulation may remain active for prolonged periods of times in seawater [49, 50] and therefore such APase could participate in different P metabolic pathways in the ocean. In future, if broad substrate specificity like PhoA is shown to exist in PhoD and PhoX, then our proposed model will be even more relevant in the marine P and C cycles.

## Conclusions

In summary, this is a case study that supports the existence of enzyme promiscuity in marine biogeochemistry and represents also an example of how a key enzyme via its promiscuity may participate in different biochemical pathways. Moreover, our results also indicate that the APase is very efficient as a monoesterase at higher concentrations. In contrast, promiscuous activities of APase, like diesterase, triesterase, and sulfatase activities, are more efficient at lower concentrations. Lastly, the uncovered promiscuous hydrolysis of P-triesters by marine APase indicates that marine microbes can cleave synthetic organophosphoesters, which serves as an example of the hidden links we are missing if the promiscuous activity of key enzymes is not considered. Collectively, this study highlights the strength of an integrative approach, combining field measurements, databases of gene sequences, and biochemical measurements of a specific enzyme to link biogeochemical function with oceanic processes. Further research is now needed since it is likely that other biogeochemically relevant promiscuous and multifunctional enzymes are widespread in the ocean [51, 52].

## Supplementary information

Supplemental Material (Figures)

Supplementary Table 1

Supplementary Table 2

Supplementary Table 3

## Data Availability

All data are available in the manuscript or the extended data.
