# Supplementary material for: Enzyme promiscuity in natural environments: alkaline phosphatase in the ocean
Source: ISME J. 2021 May 28;15(11):3375–83. doi: 10.1038/s41396-021-01013-w (PMC8528806; doi:10.1038/s41396-021-01013-w)
Supplement: Supplementary file 1 — Supplemental Material (Figures) [file 41396_2021_1013_MOESM1_ESM.docx]

**
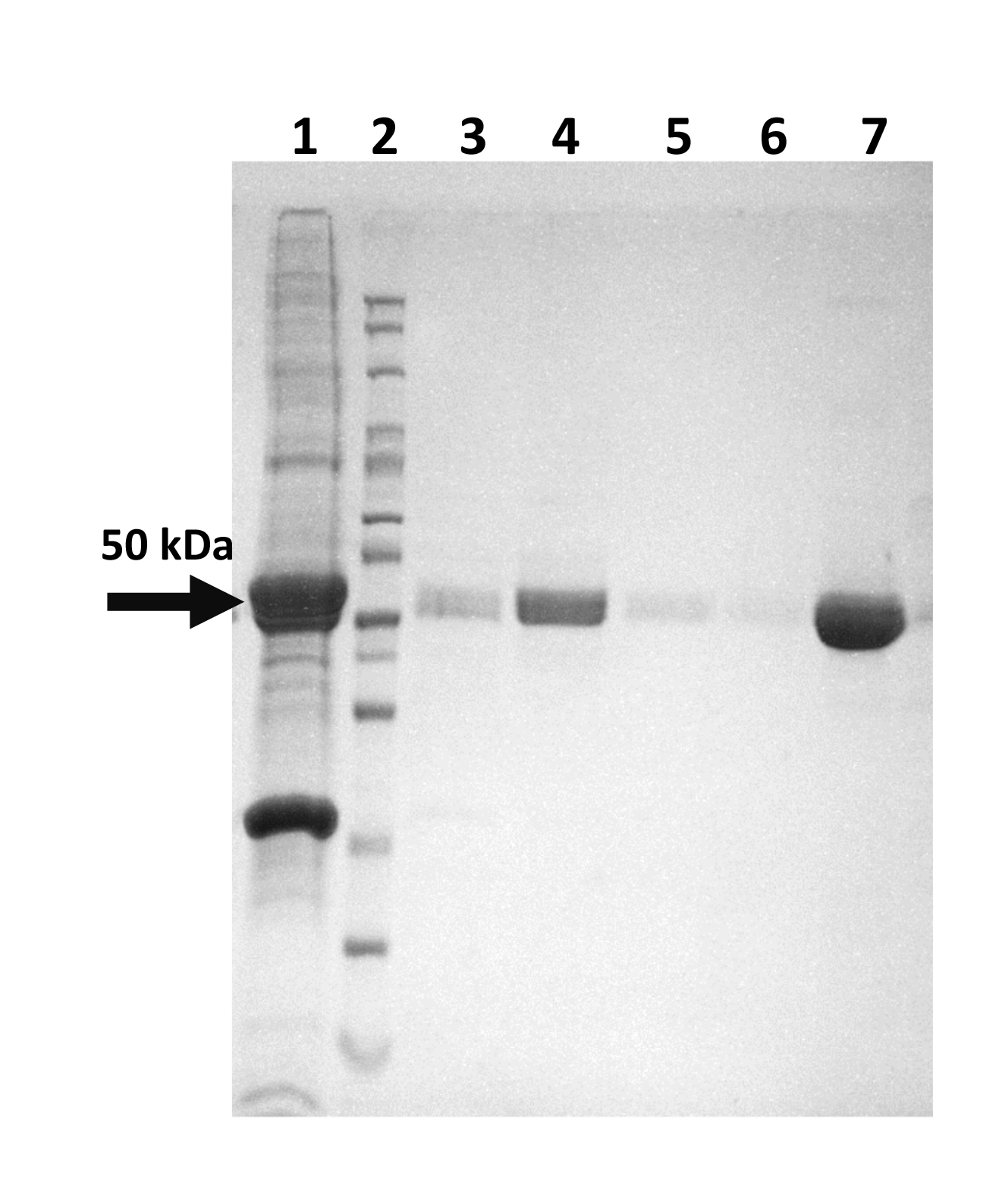
**

**Supplementary Fig. 1:** Depiction of overexpressed and the purified ~50-kDa PhoA protein. Samples were run on 12% SDS PAGE (sodium dodecyl sulfate - polyacrylamide gel electrophoresis). Black arrow indicates PhoA. Lane 1 shows whole cell lysate proteins, lane 2 is Thermo Scientific™ PageRuler™, lane 3 to 6 show elution fractions obtained from His Trap purified PhoA using 400 mM imidazole, lane 7 shows the pooled 3-6 elution fractions.


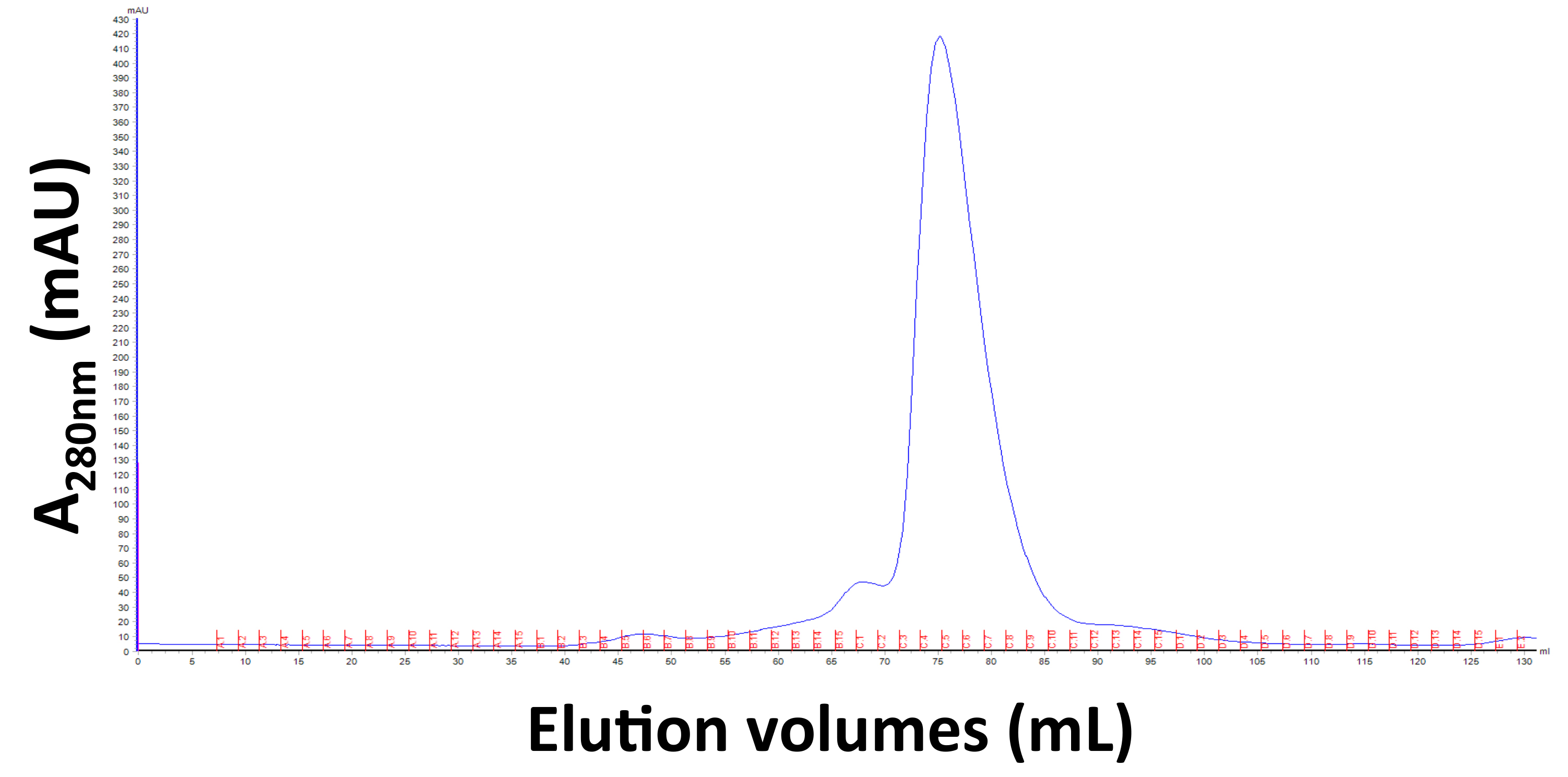


**Supplementary Fig. 2:** Chromatogram depicting purified PhoA. UV absorbance values are tabulated in Supplementary Table 3.

**
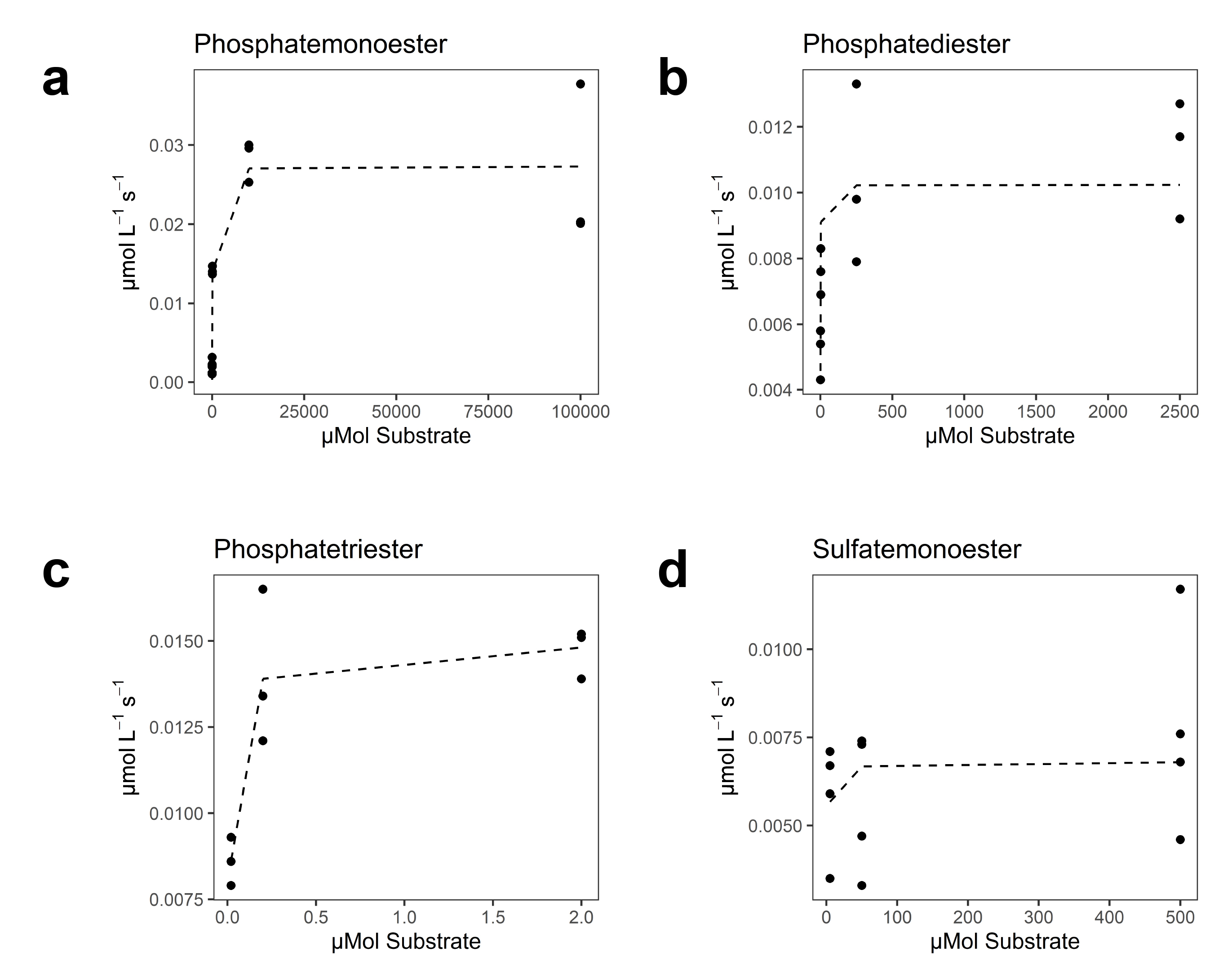
**

**Supplementary Fig. 3:** Graphs showing PhoA primary and secondary catalytic activities using variable substrate concentrations i.e. **a.** phosphomonoesterase, **b.** phosphodiesterase, **c.** phosphotriesterase, and **d.** sulfatase. Product formation was measured as a proxy of p-nitrophenol release and its detection at 405 nm.
